# The Arsenic Contamination of Drinking and Groundwaters in Bangladesh: Featuring Biogeochemical Aspects and Implications on Public Health

**DOI:** 10.1007/s00244-018-0511-4

**Published:** 2018-03-08

**Authors:** Michael Raessler

**Affiliations:** Max-Planck-Institut für Biogeochemie, Hans-Knoell-Strasse 10, PF 100164, 07745 Jena, Germany

## Abstract

Arsenic is a widespread contaminant of drinking and groundwaters in the world. Even if these contaminations have a geogenic origin, they often are exacerbated by anthropogenic activities. This is particularly true for the Bengal delta. Millions of people in Bangladesh are consuming drinking water with arsenic concentrations ≥ 50 µg/L. Their drinking water supply is based on groundwaters extracted by pumping wells, which were part of a well-drilling program by the United Nations. The intention was to provide the people with groundwater instead of surface water due to its critical hygienic conditions. Unfortunately, many wells extract the groundwater at depths where arsenic concentrations are highest. Arsenic is being dissolved from the aquifer by biogeochemical processes that are fueled by the presence of high amounts of organics in the Bengal delta sediments. This problem was not encountered at the time due to a lack of chemical analyses of the waters.

Pollution of groundwaters by arsenic is widespread throughout the globe. Very often, it is of anthropogenic origin (Raessler et al. 1999, 2000). Of particular concern in some regions, however, is the geogenic presence of arsenic in drinking water. Presumably, more than 100 million people worldwide suffer from varying amounts of arsenic in their drinking water resources. This is particularly true for Asian countries, such as Bangladesh, India, China, Mongolia, Nepal, Vietnam, Thailand, and Cambodia, but also for other regions, such as Argentina, Chile, Mexico, and parts of the United States. Additionally, European countries, such as Hungary and Romania, are concerned. The consequences are most pronounced in Bangladesh where huge parts of the population have been exposed to high amounts of arsenic for a long time (Smith et al. 2000).

Until the 1970s, people used to take surface waters from rivers and lakes for their drinking water. The tropic climate and flooding of the plain countryside during monsoon rainfall combined with hygienic deficiencies fueled the propagation of cholera and other diarrhoea diseases. To face this problem, the United Nations and UNICEF initiated a huge well-drilling program to provide the people with groundwater instead of surface water (Fig. 1). Indeed, the pumped waters were free of cholera germs. On the other hand, they often contained considerable amounts of arsenic, which was dissolved from the soils—a problem that was also true for neighbouring regions of India, i.e., the province of West Bengal. Due to a lack of chemical analyses of the waters, this problem was not encountered at the time.

**Fig. 1:**
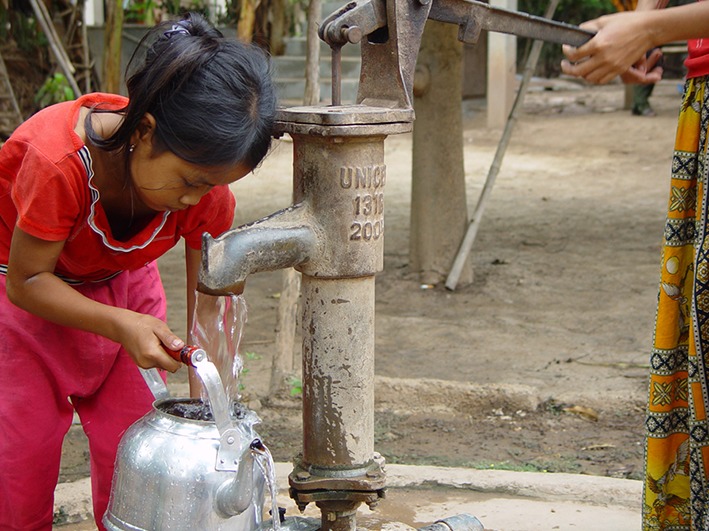
Usage of a pumping well provided by UNICEF

The pumping of groundwaters and intrusion of surface waters led to the penetration of organic matter into the groundwaters, which fueled biogeochemical processes, resulting in the dissolution of arsenic from the soil sediments (Neumann et al. 2010; Polizzotto et al. 2008).

Today, more than 3 million of 11 million wells in Bangladesh are supposed to be affected, i.e., these wells contain > 10 µg/L arsenic—a concentration that the UN recommends as a threshold value and which must not be exceeded in German drinking waters as ruled by the German drinking water directive (Trinkwasser-Verordnung 2001).

In this study, we closely look at both the hydrogeology and the biogeochemistry of arsenic in the Bengal Delta to better understand the mechanisms and the reasons for the mobility of arsenic in Bangladesh’s groundwaters and discuss the consequences for the health of the people living in these regions. Finally, recommendations to mitigate the situation will be given.

## Hydrogeology of the Bengal Delta

The Bengal Delta, most of which is part of Bangladesh, is one of the world’s largest sediment basins. It mostly consists of tertiary and quaternary sediments (Umitsu 1993; Goodbred and Kuehl 2000). These were deposited by action of the rivers Ganges, Brahmaputra, and Meghna. The different layers of the sediment are made from sand, alluvial sand, and clay minerals. The lower layers of the sediment are partly disrupted by gravels. Glacial variations of sea level led to formation of deep river gorges that were filled with new sediments. These processes strongly influenced the present properties of the aquifers and waters. The pleistocene sediment layers of the Madhupur complex are oxidized and strongly weathered offering a very good water quality. Concentrations of arsenic are mostly < 10 µg/L, in agreement with UN recommendations, although arsenic concentrations in pleistocene sediments can range up to 100 µg/L beneath deep paleo-channels at depths between 120 and 180 m (Mc Arthur et al. 2016). On the other hand, the holocenic sediments made of grey clay minerals and sand are not weathered. They often contain peat organic matter, which severely impairs water quality. These sediments were formed in a transgressive phase approximately 6000 years ago while the coastline was inundated by sea water (the sea level was approximately 2 m above today’s sea level). These holocenic sediments are highly productive and quickly renewed. Their aquifers are anoxic, favouring the mobilisation of arsenic (Smedley and Kinniburgh 2002) and characterized by high levels of calcium, magnesium, and iron (Ravenscroft et al. 2009). Both the pleistocenic and the holocenic sediments contain the most important aquifers of the Bengal delta, extending from 30 to 130 m in depth. Individual layers cannot be distinguished horizontally or vertically over longer distances. These sandy and muddy layers can be considered as aquifers of limited size. Whereas the pleistocenic aquifers are mostly free of arsenic (they serve as drinking water supply of the capital city of Dhaka), this is not the case for the holocenic aquifers, which are rich in arsenic. The highest arsenic contamination is observed in those holocenic aquifers, which are approximately 3000 years old (Dowling et al. 2002). They make up the main part of drinking water supplies in Bangladesh. The waters of the upper holocenic aquifers are approximately 100 years old and contain less arsenic. However, the holocenic layers are not homogenous and settled but are characterized by gaps and holes enabling the vertical extension of arsenic contamination. This explains the marked depth-dependence of the arsenic contamination. The highest concentrations are found in 20–70 m, which corresponds to shallow and young aquifers. On the other hand, the depth of aquifers is not a sufficient criterion for waters being free of arsenic. Moreover, it is possible that arsenic is dissolved into the waters from deeper aquifers by the pumping activity of wells for drinking water production (Mc Arthur et al. 2016).

## Biogeochemistry of Arsenic in the Bengal Delta

**Fig. 2:**
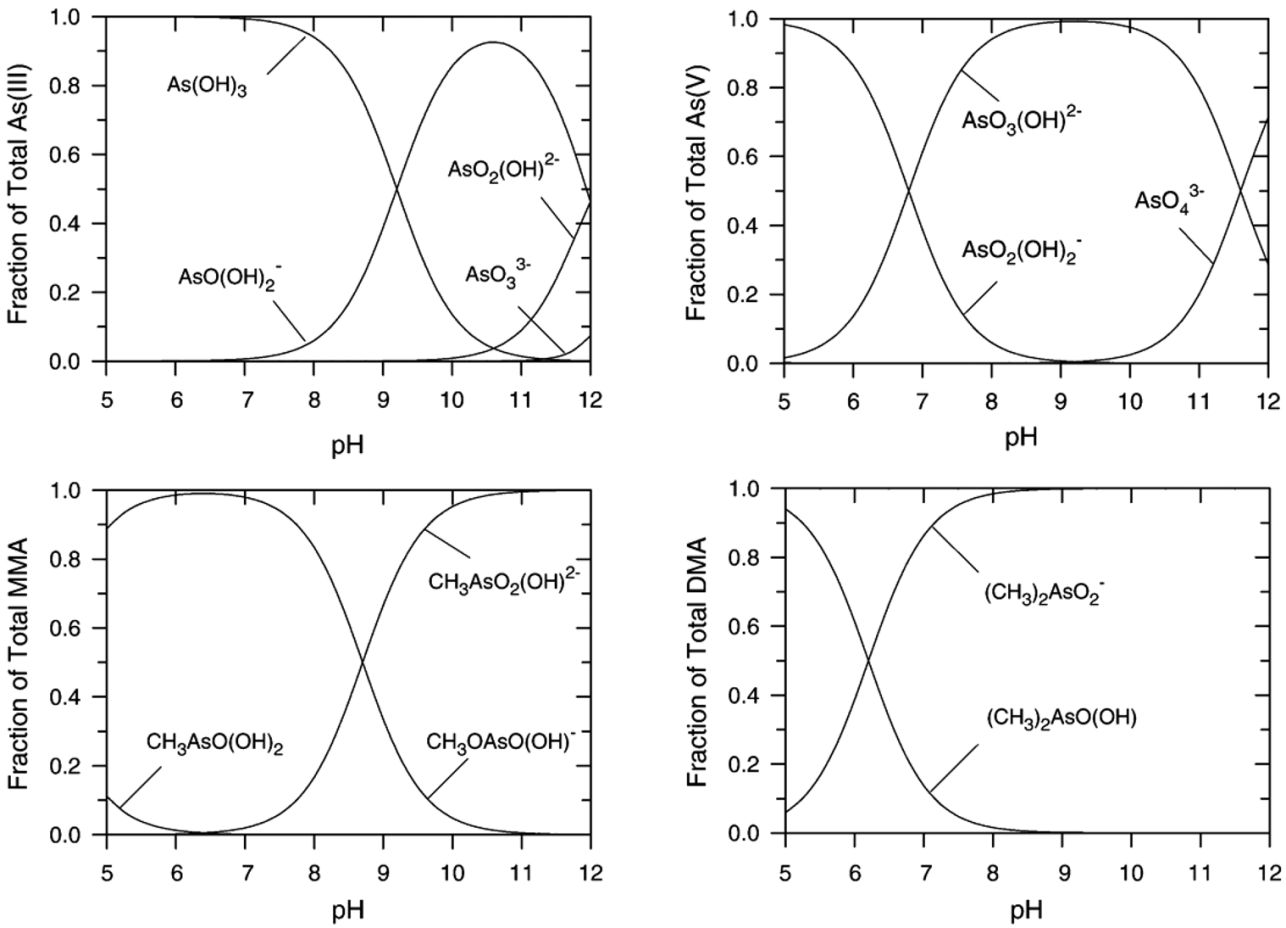
Distribution of As(III)- and As(V)-species depending on pH (Sharma and Sohn 2009)

Arsenic of geogenic origin in the Bengal delta can be dissolved from sediments into the ground waters by biogeochemical processes. Dissolution is determined by both chemical transformations and the properties of the arsenic compounds. Basically, arsenic is present in waters in two different oxidation states: As(III) (arsenious acid or arsenite) and As(V) (arsenic acid or arsenate), respectively. The degree of protonation of the arsenic species is ruled by the acidity of the water, i.e., its pH value, as shown in Fig. 2 (Sharma and Sohn 2009).

It is well known today that a variety of different factors is influencing the mobility of arsenic. Among them are chemical phenomena fostering the mobility, such as the leaching of arsenic by the carbonate ion, and others impairing its mobility, such as the formation of solid arsenic sulfide phases and also the competition of the arsenic ion with the analogous phosphate ion for sorption sites on ferric oxyhydroxides. Ravenscroft et al. (2009) distinguished the following four mechanisms of arsenic mobilisation:

- Reductive dissolution.
- Alkali desorption.
- Sulfide oxidation.
- Geothermal waters.

In the humid and tropical climate of the alluvial Bengal delta, reductive dissolution is the dominating mechanism. The primary source of arsenic contamination in Bangladesh is arsenic-bearing minerals from the Himalaya, such as arsenopyrite FeAsS, oripigment As_2_S_3_, or realgar AsS. As(III), Fe(II), and sulfate formed from sulfide had been released from the ore during weathering by action of oxygen or by oxygen dissolved in water, respectively. Within a short range of time, there was further oxidation of Fe(II) to Fe(III), which subsequently precipitated as ferric oxyhydroxide. At the same time, there was slow oxidation of As(III) to As(V), which, in turn, was adsorbed to the ferric oxyhydroxides. As(V) was thus immobilized in the sediments and did not contribute to the pollution of groundwaters. The soluble sulfate ions were eroded (Anawar et al. 2003).

Hydrochemical “on-site” studies performed in Bangladesh gave incidence that the mobility of arsenic in the groundwaters is strongly related to the availability of organic matter (Selim Reza et al. 2010). Organic carbon serves as foodstock of microorganisms able to decompose deep soil layers leading to the dissolution of arsenic (Harvey et al. 2002). It is not clear whether the organic matter originates from peat layers in the holocenic aquifers or whether it is of anthropogenic origin, caused by agriculture (fertilization), manure, waste disposal, or even measures taken for arsenic decontamination and removal, respectively. The organic matter is metabolized by the microorganisms, causing anoxic conditions by exhaustive consumption of oxygen. The ferric oxyhydroxides are reduced by action of the microorganisms, releasing Fe(II) and As(V), which is reduced to As(III). This process is characteristic of the Bengal delta and gives rise to high amounts of dissolved iron which very often correlate with As(III) concentrations. The microorganisms react in different ways with arsenic by either oxidizing arsenite or reducing arsenate (Oremland and Stolz 2005): chemoautotrophic arsenite oxidizing bacteria (CAO) oxidize dissolved As(III) to As(V) using either oxygen or nitrate as electron acceptors, while incorporating inorganic carbon (CO_2_) in the cell material. Heterotrophic arsenite oxidizing bacteria (HAO), on the other hand, use organic carbon as energy source and cell material. These processes are favoured by the constructed well systems, which will deliver the oxidative agents needed, i.e., oxygen, nitrate (Oremland and Stolz 2003). The constant supply of organic matter maintains the microbial respiration, resulting in anoxic conditions by consumption of oxygen. The anoxic conditions are used by dissimilatory arsenate respiring prokaryotes (DARP) reducing As(V) to As(III). As(V) serves them as electron acceptor during anaerobic cell respiration. DARP oxidize several organic and inorganic electron donators. Organic carbon is present in various compounds (Bisutti et al. 2004). Based on recent findings, the mobilization of arsenic is strongly fueled by the fraction of organic carbon that is biologically degradable. Biologically degradable organic carbon (BDOC) often originates from pond waters (Polizzotto et al. 2008). On the other hand, the organic carbon resulting from the irrigation of paddy fields is mostly recalcitrant and will not be metabolized by the microorganisms. Consequently, this organic carbon does not contribute to arsenic mobilization. This hypothesis is supported by relatively low arsenic contents found in ground waters from paddy fields. Moreover, incubation and column experiments with pond waters after action of oxygen led to a reasonable decrease in organic carbon which was not observed with organic carbon of paddy fields ground waters (Neumann et al. 2010). The impact of BDOC on the mobilization of arsenic also was observed in sediments of the Mekong delta in Cambodia (Seyfferth et al. 2014). This is of particular interest, as both the geological and anthropogenic settings are quite different. Due to the much higher population density in Bangladesh, anthropogenic influences do have a much greater impact than in the Mekong delta. Further and intense research activities will be necessary to clarify the relationship between the nature and the impact of organic carbon on the mobilization and thus the biogeochemistry of arsenic in Bangladesh and other contaminated regions.

## The Toxicity of Arsenic

The toxicity of arsenic has been known for centuries. Prominent examples are the arsenic poisonings of Napoleon Bonaparte and Descartes, as well as the German murderer Gesche Gottfried who was executed in Bremen in 1831 for several crimes committed by diarsenic trioxide As_2_O_3_. The lethal dose for ingested As_2_O_3_ in humans is 70–180 mg (Léonard 1991).

**Fig. 3:**
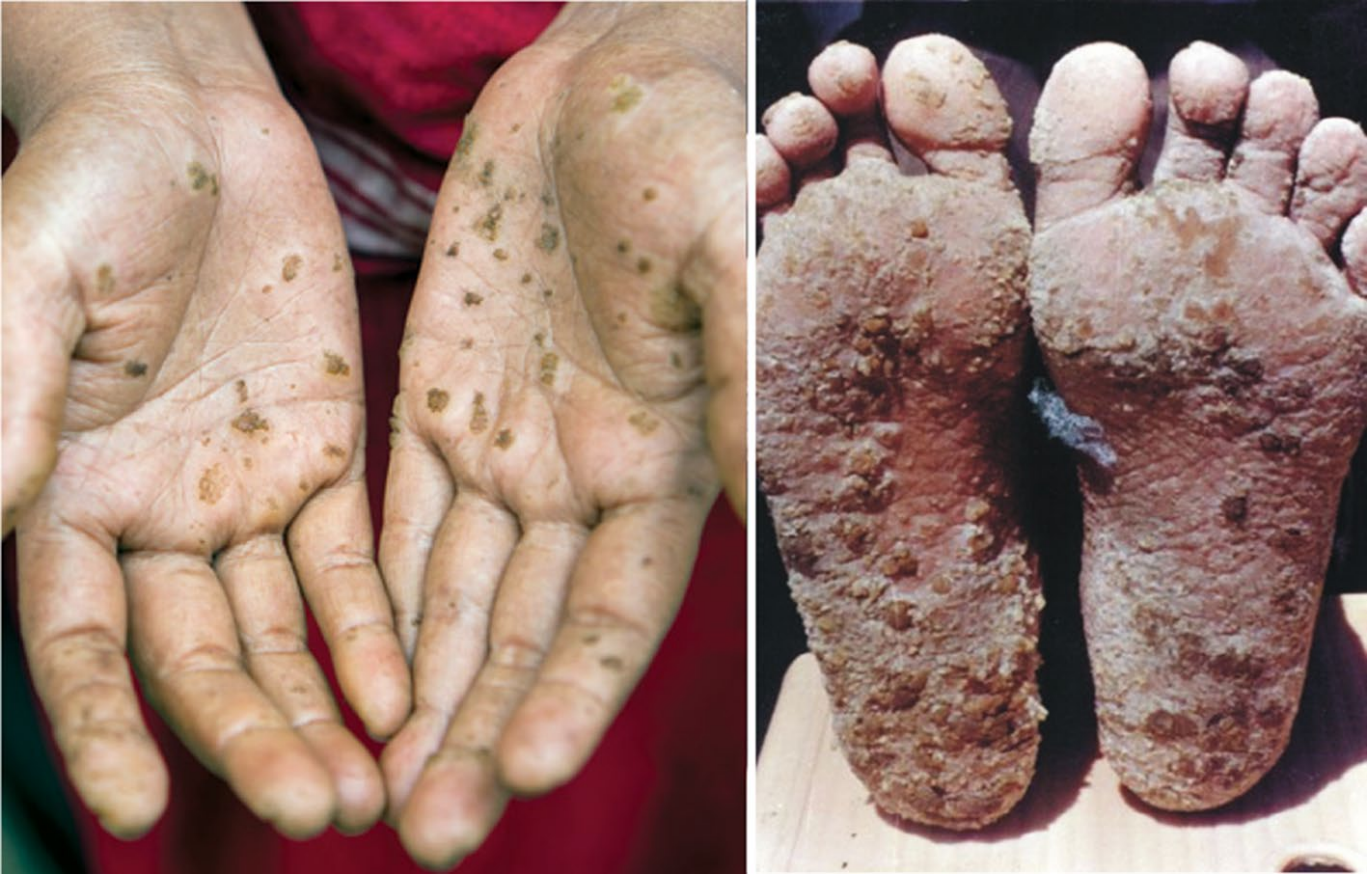
Nodes on hands and soles of the foot caused by arsenic poisoning (keratosis)

The consequences of longer exposer to arsenic are numerous. Arsenic is a capillary and enzyme poison due to its high affinity for sulfur-containing proteins. Chronic arsenic poisoning—arsenicosis—often is accompanied by a keratosis, which is a painful skin disease with eczemas and ulcers. Inorganic arsenic compounds originate the formation of the characteristic nodes on the hands and soles of the foot (shown in Fig. 3).

Even worse are skin maligna (Guha Mazumder et al. 1998). Additionally, senso- and vaso-motoric disorders are observed, which can result in a collapse of the patient.

Both mobility and accumulation of arsenic strongly depend on the presence of its compounds in the environment, i.e., its chemical speciation. Examples are the inorganic compounds arsenite [As(III)] and arsenate [As(V)], as well as the organic compounds monomethlylarsenite [MMA(III)], monomethlylarsenate [MMA(V)], dimethylarsenite [DMA(III)], and dimethylarsenate [DMA(V)]. The latter is transformed from the inorganic arsenic compounds by biomethylation (Cullen and Reimer 1989). The order of toxicity of the compounds is as follows: MMA(III) > DMA(III) > As(III) > As(V) > MMA(V) > DMA(V).

## Groundwater Contamination and Implications for Human Health

The problem of groundwater contamination by arsenic also concerns many other people in developing countries in South East Asia where nonmonitored groundwaters serve as the main source of drinking water supply.

### The Arsenic Contamination of Rice

The arsenic contamination of rice, which is the most important feedstock of the region, has become more severe (Meharg et al. 2009). Rice is an important path of ingestion of inorganic arsenic compounds into the human organism. The rice plant takes up the arsenic from the soil to accumulate into its grain. The arsenic contamination of the rice plant is not only threatening human beings but also to the cattle that often are fed with rice straw. The high rice consumption in South East Asia has become the preponderant arsenic source for people not suffering from contaminated groundwaters. The average daily rice consumption for an adult in Bangladesh ranges from 400 to 650 g. This is one of the highest per capita rice consumption figures in the world (Joseph et al. 2015; Ahsan and Del Valls 2011). Irrigation of rice fields is mandatory in the dry season, also called “boro”. It was estimated that almost 1000 metric tons of As were cycled with irrigation water during the dry season each year due to the fact that boro rice needs huge amounts of water (Saha and Ashraf Ali 2007). In a recent study, more than 900 polished rice samples collected from several districts of Bangladesh were analyzed for their arsenic content. While mean and median values of total arsenic concentrations were 126 and 107 µg/kg, respectively, a maximum concentration of arsenic of 680 µg/kg, dry weight, was measured (Islam et al. 2017).

In 2014, the World Health Organization (WHO) recommended a maximum level for arsenic of 0.2 mg/kg rice. Under anaerobic conditions such as those found in flooded rice paddies, arsenite predominates which may cause severe problems. The rice plant is unable to distinguish between arsenite and silicic acid. In general, silicon is beneficial to the rice plant as it strengthens the rice plant’s stem and protects it against pathogens. It is noteworthy that rice can accumulate 10 times more silicon than other grasses which means that rice also can accumulate 10 times more arsenic. In case of aerobic conditions, arsenic will mostly be present as arsenate, which cannot be distinguished from phosphate by the rice plant, which is an important nutrient to it. Currently, the arsenic accumulation of different rice cultivars is examined. Those cultivars revealing lowest accumulation of arsenic could be used as stock for breeding programs. On the contrary, rice cultivars that accumulate less arsenic also will accumulate less silicon and, consequently, will be less stable and more prone to diseases. An easier way to reduce the arsenic content in rice seems to change the way rice is boiled. Using excess water to cook rice and pouring off the extra water decreases the amount of arsenic in cooked rice. Using an off-the-shelf coffee percolator can remove up to 85% of arsenic from the rice (Carey et al. 2015) and up to 96% of arsenic from rice bran (Signes-Pastor et al. 2017).

### Other Risks of Arsenic Contamination

There are not many data available with respect of arsenic contamination of milk and meat. In Bangladesh, cattle are generally fed by rice straw and rice husk, respectively. There is almost no grassing land for cattle due to the high population density. Cattle also are exposed to contaminated water; however, dairy cows typically access surface water in Bangladesh. Consequently, arsenic concentrations detected in cow milk are generally low (Ghosh et al. 2013). However, there seems to be the risk of adultering the raw milk by diluting it with contaminated water. Due to the limited number of studies from Bangladesh and, more importantly, the limited sample sizes in almost all of these studies, a conclusive species-specific observable range for arsenic in edible plant parts (except rice) and animal-origin food cannot be defined as yet (Joseph et al. 2015).

This also applies to the data on the arsenic content of breast milk samples of women from Bangladesh. Arsenic content was found to be low (Islam et al. 2014) and consequently, exclusive breast-feeding of infants was considered to be a protection against arsenic exposure (Fängström et al. 2008).

However, this conclusion suffers from very limited data. Moreover, the situation is worsened by the fact that the number of studies on arsenic in human breast milk samples is generally much lower than compared with other elements, such as cadmium or mercury, where many more data are available. With respect to Bangladesh, meaningful data on a possible contamination of human breast milk are almost completely missing (Rebelo and Caldas 2016).

## Outlook and Recommendations

Although the problem of arsenic contamination of ground and drinking waters in Bangladesh came to public attention more than two decades ago, it is far from being resolved. According to a new report by Human Rights Watch released on April 6, 2016, in 2013 approximately 12% of rural households, which corresponds to approximately 20 million people in Bangladesh, were still consuming waters with arsenic concentrations > 50 µg/L. Although arsenic occurs naturally in Bangladesh’s groundwater, the deadly contamination of the drinking water of many millions of Bangladesh’s rural poor is a disaster that humans have caused and perpetuated. The same report states that the government of Bangladesh is failing to address this predicament of the rural poor, but on the contrary, is expending considerable resources in areas where the risk of arsenic contamination is relatively low and where water coverage is relatively good. It provides evidence of how the positioning of tube wells can sometimes represent a political manoeuvre by government officials keen to garner support, rather than the prioritization of safe water provision to the areas of greatest risk of arsenic contamination.

Despite government reports stating that the government should do a better job of targeting arsenic mitigation options in areas where there are most needed, it inexplicably fails to do so. Additionally, there is a serious lack of monitoring and quality control in arsenic mitigation projects. Although in 2005, the country adopted a “pro-poor strategy” to prioritize poor villagers when new water points were allocated, there was no substantial improvement of the situation. Public awareness campaigns stressing the dangers of arsenic ended many years ago. The government’s approach to well water testing, which relies on villagers bringing their water samples to an office, does not work well. Moreover, within the health system, the impact of past and current exposure to arsenic on people’s health is being largely ignored.

Consequently, there is a need to improve targeting of high-priority areas and to end the pernicious influence of political representatives on the allocation of new water points. The report also asks for increasing care by international donors, such as UNICEF or the World Bank, which support Bangladesh’s government efforts to install safe water points, when executing and monitoring projects. This includes remediation plans for communities serviced by contaminated water points, which means that wells with arsenic contents above the threshold value are closed or rehabilitated, respectively.

Massive efforts are needed to test all wells, including private, shallow tubewells. This could be done by distribution of so-called “penny per test” kits, which are under development of the NGO “Chemists Without Borders” (C&EN 2016). In addition, development and application of measures for arsenic remediation have to be intensified (Sarkar and Paul 2016; Singh et al. 2015).

Another important issue to be tackled is the lack of sufficient and reliable data on arsenic contamination of major foodstuffs in Bangladesh, i.e. rice, legumes, milk, and dairy products. So far, it is almost impossible to draw serious conclusions. Of course, this also is true for data on arsenic contamination of the “rural poor”, i.e., those people of Bangladesh who are too poor to own their own land who live in rural villages in areas with elevated arsenic groundwater levels. Screening of arsenic data of body fluids, such as blood, urine, and breast milk, which implies that people suspected of having arsenic-related health impairments would have access to medical treatment, is mandatory. Until all of Bangladesh’s people have access to safe water, their right to health will remain denied.
